# Postnatal Development of Microglia-Like Cells in Mouse Cochlea

**DOI:** 10.1155/2018/1970150

**Published:** 2018-07-31

**Authors:** Penghui Chen, Yongchuan Chai, Haijin Liu, Gen Li, Longhao Wang, Tao Yang, Hao Wu

**Affiliations:** ^1^Department of Otorhinolaryngology-Head and Neck Surgery, Shanghai Ninth People's Hospital, Shanghai Jiao Tong University School of Medicine, Shanghai, China; ^2^Ear Institute, Shanghai Jiao Tong University School of Medicine, Shanghai, China; ^3^Shanghai Key Laboratory of Translational Medicine on Ear and Nose Diseases, Shanghai, China; ^4^Department of Pediatric Surgery, The First Affiliated Hospital of Gannan Medical University, Jiangxi Province, China

## Abstract

Microglial cells are involved in surveillance and cleaning of the central nervous system. Recently, microglial-like cells (MLC) have been found in an adult cochlea and investigated for their role in cochlear inflammation. The presence and potential roles of MLCs during the development of the cochlea, however, remain unclear. In this study, immunostaining was performed using the MLC-specific marker IBA1 to characterize the presence, distribution, and morphology of MLCs in the developing cochlea. From P0 to P14, MLCs were present in a variety of cochlear regions including the modiolus, spiral lamina, spiral ganglion, spiral ligament, and the organ of Corti. Interestingly, the overall number of MLCs in a mouse cochlea steadily increased since P0, peaks at P5, then gradually decreased from P5 to P14. In the spiral ligament, the distribution of the MLCs trends to shift from the type I/II fibrocyte-rich regions to the type III/IV fibrocyte-rich regions during the course of cochlear development, accompanied by the morphological changes of MLCs from the amoeboid, activated form to the ramified, quiescent form. Our results suggested that MLCs experience drastic morphological and distributional changes during postnatal cochlear development, which may play a role in the maturing and remodeling of the cochlea.

## 1. Introduction

Microglia-like cells (MLC) are bone marrow-derived cells that act as tissue-resident macrophage in the inner ear. The cochlear labyrinth was used to be thought of as immunoprivileged, free of macrophage. More recently, increasing studies demonstrated the existence of both resident cochlear macrophages and recruitment of inflammatory macrophages to the cochlea [1–4].

Observation of MLCs has been originally reported in the avian and murine inner ear [5, 6]. In 2008, Okano et al. found that bone marrow-derived cells expressing the microglia-specific marker IBA1 are present as tissue-resident macrophages in the mouse inner ear [1, 3]. Recently, O'Malley et al. reported the existence of IBA1+, CD68+, and CD163+ macrophages/microglia in the adult human cochlea [7]. Seigel et al. isolated and enriched the population of CD11b+ cells from the cochlea and immortalized these cells to derive a novel microglial cell line named Mocha (microglia of the cochlea) [8].

Microglial cells function as the resident immune cells of the central nervous system (CNS), retina, and inner ear and are primary mediators of inflammations [9]. Many studies indicate that excessive microglial activation is deleterious for the neuron, while microglial inhibition may reduce neural damage. In the inner ear, deactivation of MLC minimizes hair cell loss and improves hearing after cochlear damage [10, 11]. However, other studies showed that microglial activation may also have some neuroprotective effects [6].

Microglia in the CNS originates from cells of mesodermal lineage. In the cochlea, the MLCs originate from bone marrow-derived cells [2, 4]. To date, the distribution of MLCs was mostly studied in the adult cochlea, where the presence of MLCs was detected in the stria vascularis, spiral ligament, basilar membrane, and 8th nerve [1, 7]. On the other hand, the microglial morphology and distribution during cochlear development have been far less investigated. This paper aimed at exploring the origins and distribution of the resident MLCs in the mouse cochlea during development.

## 2. Materials and Methods

### 2.1. Animals

C57BL/6 mice were bought from Shanghai SLAC Laboratory Animal Co. All animal procedures were performed following protocols that were approved by the Institutional Authority for Laboratory Animal Care of Ninth People's Hospital, School of Medicine, Shanghai Jiao Tong University. All possible efforts were made to minimize the number of animals used and their suffering. In our experiments, P0 was defined as the day of birth.

### 2.2. Immunohistochemistry

Cochleae were quickly extracted and immersion fixed in 4% paraformaldehyde overnight at 4°C. Decalcification was performed for cochleae of P7 and later for 24 hours. The specimens were then dehydrated with a graded series of ethanol and embedded in paraffin wax. Paraffin sections in 3 um thickness were deparaffinized, rehydrated, antigen retrieved, and blocked with 10% normal rabbit serum at room temperature for 30 min. Slides were incubated with a primary antibody (IBA1, Abcam, Ab178846 1 : 500 diluted with PBS) overnight at 4°C, washed three times with PBS (pH 7.4) for 5 min each, and then incubated with a secondary antibody (secondary antibody: HRP-goat anti-rabbit 1 : 300) labelled with HRP at room temperature for 50 min. Freshly prepared DAB chromogenic reagent was added to the dry sections. Nuclei were stained blue with hematoxylin while the nuclei of IBA1+ cells were stained brown-yellow with DAB reagent.

### 2.3. Transmission Electron Microscopy (TEM)

Cochleae were perfused with ice-cold 2.5% glutaraldehyde and immersion fixed overnight at 4°C. After postfixing in 2% osmium tetroxide at 4°C for 1 h, cochleae were dehydrated and embedded in 812 resin. The ultrathin sections were stained with lead citrate and uranyl acetate and observed under a Philips CM-120 transmission electron microscope (Philips, Amsterdam, Netherlands).

### 2.4. Quantification and Statistical Analysis

**Figure 1:**
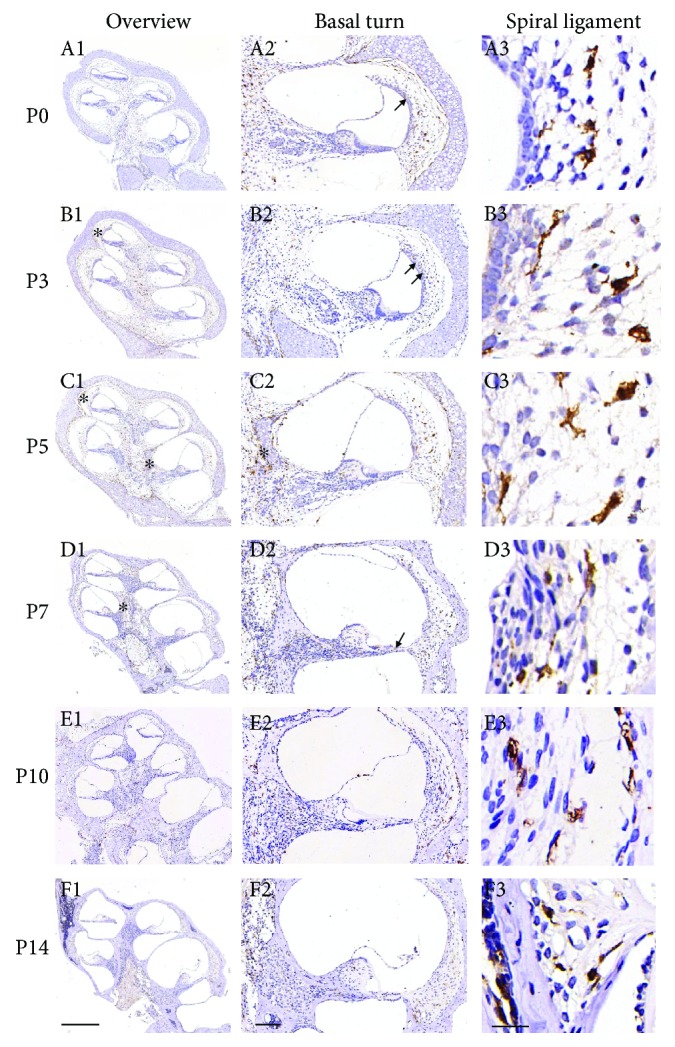
Distribution and morphology of IBA1+ cells in the postnatal development of a mouse cochlea. Left column: overview, 5x, scale bar = 400 um. Middle column: basal turn, 10x, scale bar = 100 um. Right column: spiral ligament at basal turn, 90x, scale bar = 20 um. Rows A–F: P0, P3, P5, P7, P10, and P14, respectively. Arrows: IBA1+ cells on the undersurface of the basilar membrane in the scala tympani and the stria vascularis. Asterisks: IBA1+ cells in the endosteal layer of the cochlea.

The number of IBA1+ cells was counted for the overall section of the cochlea (Figure 1, left column) and for sections of the basal turn lateral wall (Figure 1, right column) in three continuous immunohistochemistry slices. The statistical significance of different IBA1+ cell numbers among adjacent observation time points was analyzed using Student's *t*-test. *P* values of 0.05 or less were deemed as statistically significant.

## 3. Results

### 3.1. Presence of MLCs in Mouse Cochlea during Development

**Figure 2:**
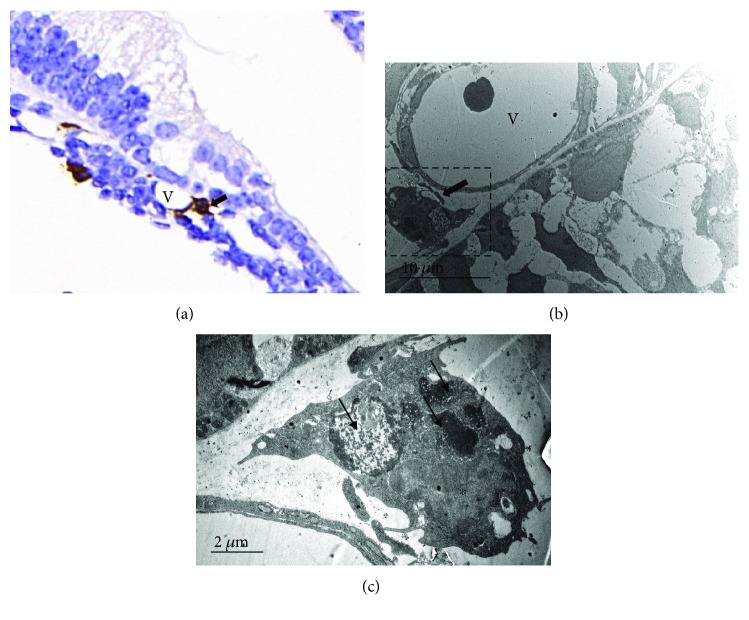
Perivascular resident MLC at basal membrane of P3 mouse cochlea. (a) Immunohistochemistry images. Arrow: MLC; V: vessel. (b) TEM images. Arrow: MLC; V: vessel. (c) Magnification of the dashed box of B. Asterisk: nuclei of MLC. Arrows: engulfing and digesting of the cellular debris.

In P0–P14 mouse cochleae, IBA1+ cells were mainly observed in the modiolus, spiral lamina, spiral ganglion, spiral ligament, and the organ of Corti (Figure 1). Spots of IBA1+ cells were detected on the undersurface of the basilar membrane in the scala tympani and the stria vascularis (Figure 1, arrows) and were abundantly present at the endosteal layer of the cochlea, especially at the early postnatal stages (Figure 1, asterisks). At P5, the IBA1+ cells clustered at the modiolus around the cartilage (Figure 1, C2), which disappeared after P7. Some IBA1+ cells can be occasionally identified in close vicinity to the large vessels of the organ of Corti (Figure 2).

**Figure 3:**
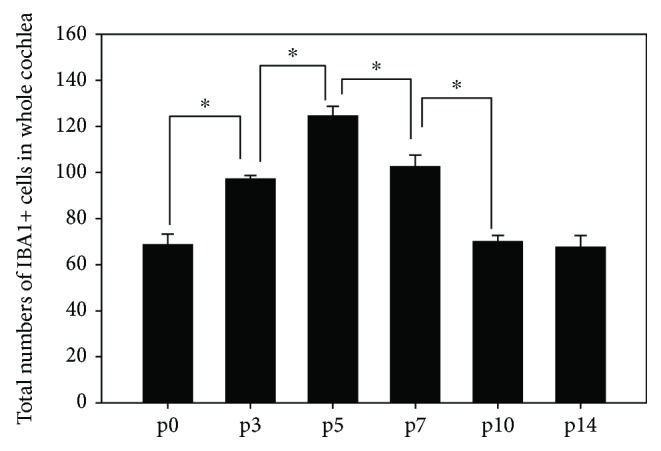
Number counts of IBA1+ cells at midmodiolar sections of P0–P14 mouse cochleae. Asterisks: statistical significance (*P* < 0.05).

The total number of IBA1+ cells changed significantly during the postnatal development of the mouse cochlea, increasing from P1 to P5 by nearly 2-fold and then decreased to the original level from P5 to P14 (Figures 1 and 3).

### 3.2. Distribution and Morphology Change of MLCs in the Spiral Ligament of Mouse Cochlea during Development

In this study, we observed a significant change of the distribution and morphology of the MLCs in the spiral ligament during the development of the mouse cochlea. At P0, MLCs mainly inhabited the peripheral and central regions of the spiral ligament that are filled with type I and type II fibrocytes. From P0 to P14, MLCs gradually migrated towards the subcentral region and marginal region that are filled with type III and IV fibrocytes (Figure 1, right column; see Supplementary Figure 1 for the localization of type I, II, III, and IV fibrocytes in the lateral wall of the cochlea).

A significant morphological change of the MLCs was also observed in the spiral ligament during mouse cochlear development. Representative shapes of the different types of MLCs are shown in Supplementary Figure 2. At P0, most MLCs in the spiral ligament were in a round, amoeboid shape (Figure 1, A3), indicative of the activated form of MLCs [12]. At P3, some extended, bipolar-like MLCs appeared in the spiral ligament (Figure 1, B3) and at P5 this form of MLCs became the majority (Figure 1, C3). At P7, the MLCs were further extended and arborized (Figure 1, D3). At P14, most MLCs have a ramified shape, characterized by long branching processes and a small cellular body (Figure 1, E3) and indicative of the quiescent form of MLCs.

## 4. Discussion

The current study investigated the presence, distribution, and morphology of MLCs in a developing mouse cochlea. Consistent with previous findings in the adult mouse cochlea [1, 7], MLCs were identified in similar regions including the modiolus, spiral lamina, spiral ganglion, spiral ligament, and the organ of Corti from P0 to P14 (Figure 1). The abundance, distribution, and morphology of MLCs, however, underwent a significant change during the postnatal development of the cochlea.

In the current study, we observed a change of the overall abundance of MLCs in the mouse cochlea between P0 and P14. The total number of the IBA1+ cells steadily increased from P0 to P5 and then decreased back to the original level from P5 to P14 (Figure 3). In the CNS, microglia play a critical role in tissue homeostasis and innate immunity by constantly removing infectious agents or unwanted cells. Activated microglial cells are also capable of producing both neurotrophic factors and antioxidants that can improve the neuronal function as well as neurotoxic cell signal factors [12–15]. Between P0 and P14, as the hearing function is gradually maturing, a great variety of cells in the cochlea will undergo different cell fates [16, 17]. Some cells such as glial cells and supporting cells may undergo extensive differentiation and proliferation, while other cells in regions such as the greater epithelial ridge (GER) and the lesser epithelial ridge (LER) may enter programmed apoptosis [16]. Therefore, it is conceivable that during the postnatal cochlear development, MLCs may assist in cleaning and removing the dead or dying cells and its abundance may change accordingly during the process.

In the spiral ligament of the developing cochlea, we also observed a significant change of the distribution and morphology of MLCs. Between P0 and P14, MLCs migrated from the type I/II fibrocyte-rich regions to the type III/IV fibrocyte-rich regions with a morphological shift from the amoeboid, activated form to the ramified, quiescent form (Figure 1). In the CNS, ramified, resting microglia surveys and assesses the microenvironment by the extension and retraction of its processes. In response to physiological changes, it can de-ramify into the phagocytic amoeboid form [9]. The spiral ligament of the cochlea consists of connective tissue cells, epithelial cells, blood vessels, and extracellular matrix material. We speculated that MLCs may help in remodeling the extracellular matrix of collagen fibers at different developmental stages.

Based on our observations, we speculated that for cells entering programmed apoptosis during postnatal cochlear development, removal of dead or dying cell debris may require the assist of activated MLCs. Accordingly, the disruption of MLCs may interfere with the normal development and maturing of the mouse cochlea. Due to limited technical solutions to directly deactivate the MLCs, however, it is difficult to prove this point experimentally at the current stage. A drug specifically targeting the MLCs in the developmental cochlea is needed for this purpose.

## 5. Conclusions

MLCs are present in the developing mouse cochlea and change in distribution and morphology during the process, suggesting a key role in cochlear development.

## Data Availability

Readers can access additional experimental data in optional supplementary materials.

## Supplementary Materials

Supplementary 1 Supplementary Figure 1: localization of type I, II, III, and IV fibrocytes in the lateral wall of the cochlea.

Supplementary 2 Supplementary Figure 2: representative shapes of the different types of MLCs. (A) round, amoeboid; (B) extended, bipolar-like; (C) further extended and arborized; and (D) ramified.
