# Supplementary figures and images for: Postnatal Development of Microglia-Like Cells in Mouse Cochlea
Source: Neural Plast. 2018 Jul 31;2018:1970150. doi: 10.1155/2018/1970150 (PMC6091412; doi:10.1155/2018/1970150)

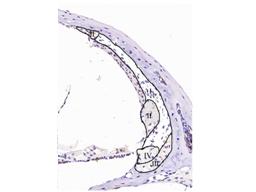

Supplement: Supplementary 1 — Supplementary Figure 1: localization of type I, II, III, and IV fibrocytes in the lateral wall of the cochlea. [file 1970150.f1.xps › docProps/thumbnail.jpeg]

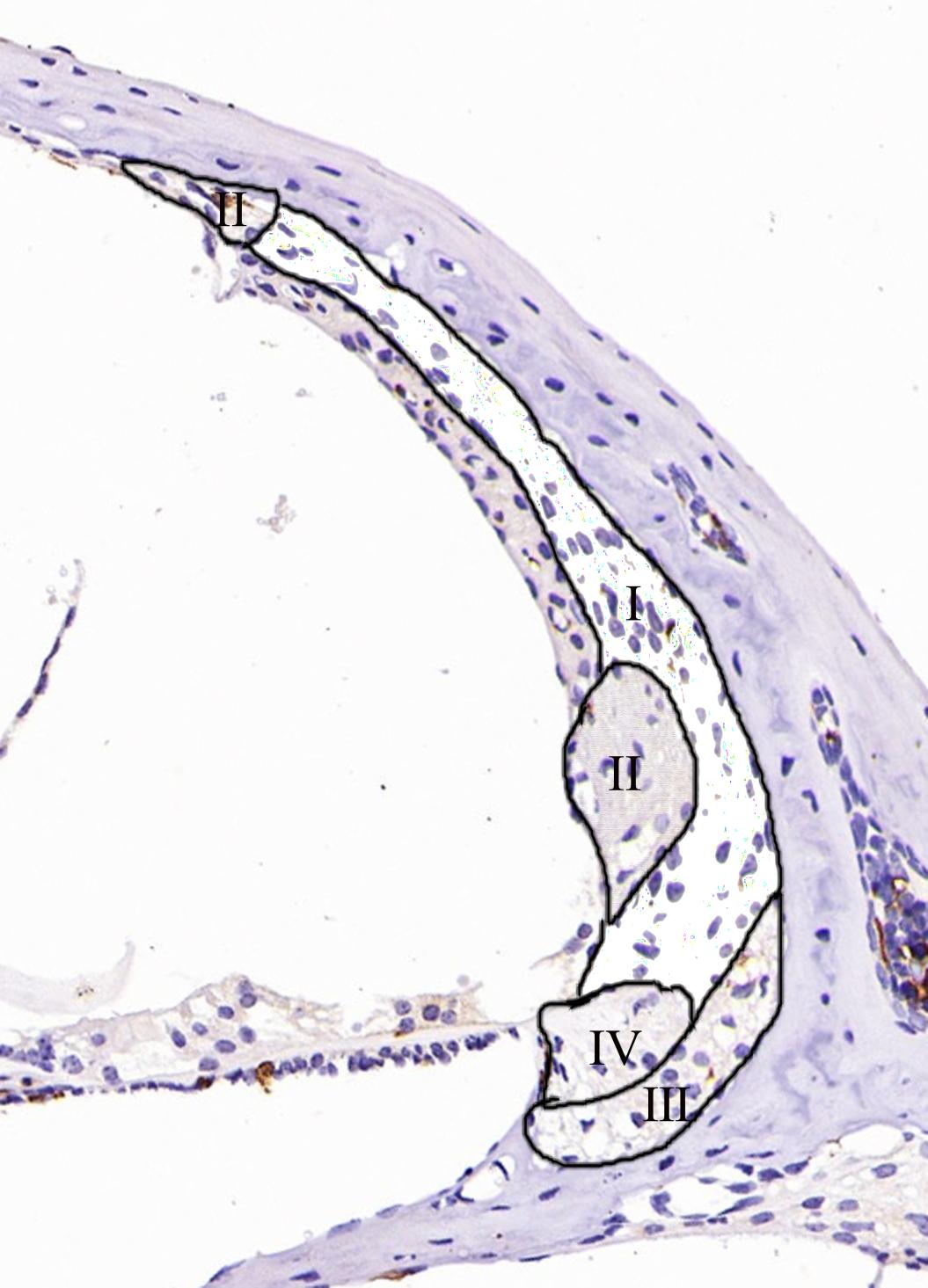

Supplement: Supplementary 1 — Supplementary Figure 1: localization of type I, II, III, and IV fibrocytes in the lateral wall of the cochlea. [file 1970150.f1.xps › Resources/Images/image_0.jpg]

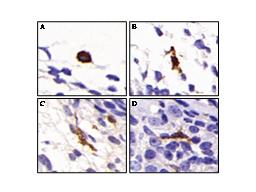

Supplement: Supplementary 2 — Supplementary Figure 2: representative shapes of the different types of MLCs. (A) round, amoeboid; (B) extended, bipolar-like; (C) further extended and arborized; and (D) ramified. [file 1970150.f2.xps › docProps/thumbnail.jpeg]

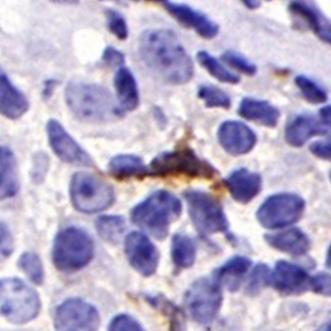

Supplement: Supplementary 2 — Supplementary Figure 2: representative shapes of the different types of MLCs. (A) round, amoeboid; (B) extended, bipolar-like; (C) further extended and arborized; and (D) ramified. [file 1970150.f2.xps › Resources/Images/image_3.jpg]

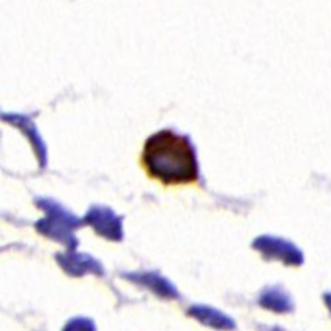

Supplement: Supplementary 2 — Supplementary Figure 2: representative shapes of the different types of MLCs. (A) round, amoeboid; (B) extended, bipolar-like; (C) further extended and arborized; and (D) ramified. [file 1970150.f2.xps › Resources/Images/image_0.jpg]

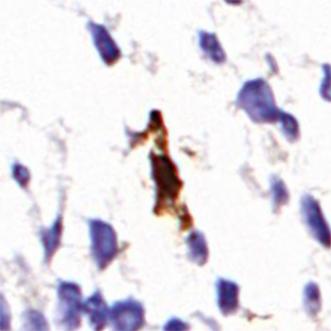

Supplement: Supplementary 2 — Supplementary Figure 2: representative shapes of the different types of MLCs. (A) round, amoeboid; (B) extended, bipolar-like; (C) further extended and arborized; and (D) ramified. [file 1970150.f2.xps › Resources/Images/image_1.jpg]

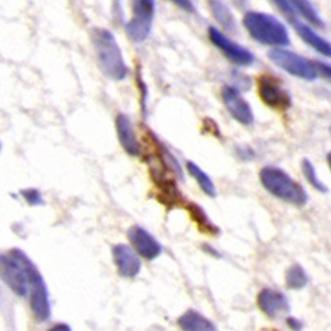

Supplement: Supplementary 2 — Supplementary Figure 2: representative shapes of the different types of MLCs. (A) round, amoeboid; (B) extended, bipolar-like; (C) further extended and arborized; and (D) ramified. [file 1970150.f2.xps › Resources/Images/image_2.jpg]
